# Machine Learning Model for Predicting Pathological Invasiveness of Pulmonary Ground‐Glass Nodules Based on AI‐Extracted Radiomic Features

**DOI:** 10.1111/1759-7714.70128

**Published:** 2025-07-31

**Authors:** Guozhen Yang, Yuanheng Huang, Huiguo Chen, Weibin Wu, Yonghui Wu, Kai Zhang, Xiaojun Li, Jiannan Xu, Jian Zhang

**Affiliations:** ^1^ Department of Cardiothoracic Surgery Third Affiliated Hospital of Sun Yat‐sen University Guangzhou China

**Keywords:** artificial intelligence, invasiveness, pulmonary ground‐glass nodules, radiomics

## Abstract

**Background:**

With the widespread adoption of low‐dose CT screening, the detection of pulmonary ground‐glass nodules (GGNs) has risen markedly, presenting diagnostic challenges in distinguishing preinvasive lesions from invasive adenocarcinomas (IAC). This study aimed to develop a machine learning (ML)–based model using artificial intelligence (AI)‐extracted CT radiomic features to predict the invasiveness of GGNs.

**Methods:**

A retrospective cohort of 285 patients (148 with preinvasive lesions, 137 with IAC) from the Lingnan Campus was divided into training and internal validation sets (8:2). An independent cohort of 210 patients (118 with preinvasive lesions, 92 with IAC) from the Tianhe Campus served as external validation. Nineteen radiomic features were extracted and filtered using Boruta and LASSO algorithms. Seven ML classifiers were evaluated using AUC‐ROC, decision curve analysis (DCA), and SHAP interpretability.

**Results:**

Median CT value, skewness, 3D long‐axis diameter, and transverse diameter were ultimately selected for model construction. Among all classifiers, the Gradient Boosting Machine (GBM) model achieved the best performance (AUC = 0.965 training, 0.908 internal validation, and 0.965 external validation). It demonstrated strong accuracy (88.1%), specificity (80.7%), and F1 score (0.87) in the external validation cohort. The GBM model demonstrated superior net clinical benefit. SHAP analysis identified median CT value and skewness as the most influential predictors.

**Conclusion:**

This study presents a simplified ML model using AI‐extracted radiomic features, which has strong predictive performance and biological interpretability for preoperative risk stratification of GGNs. By leveraging median CT value, skewness, 3D long‐axis diameter, and transverse diameter, the model enables accurate and noninvasive differentiation between IAC and indolent lesions, supporting precise surgical planning.

## Graphical abstract

A retrospective cohort of 495 patients with pathologically confirmed GGNs (AAH/AIS/MIA: 266; IAC: 229) from two centers was analyzed. AI‐extracted CT radiomic features (*n* = 19) were processed via a two‐stage feature selection (Boruta algorithm + LASSO regression). Seven ML models were trained/validated using an 8:2 internal split and external cohort (*n* = 210).

Four features were selected: median CT value, skewness, 3D long diameter, and transverse diameter. The Gradient Boosting Machine (GBM) model achieved superior performance:Training cohort (*n* = 285):

AUC 0.965 (95% CI: 0.944–0.985)

- Internal validation (*n* = 114): AUC 0.908 (95% CI: 0.824–0.992)
- External validation (*n* = 210): AUC 0.965 (95% CI: 0.945–0.984)
- SHAP analysis confirmed median CT value as the top predictor (*p* < 0.001)
- Decision curve analysis demonstrated clinical utility

An AI‐derived radiomic model accurately stratifies GGN invasiveness using four CT features. Its high performance in multi‐center validation supports integration into preoperative workflows to personalize surgical management.

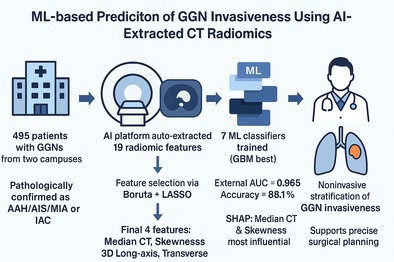

## 1 Introduction

With the increasing adoption of low‐dose computed tomography (LDCT), the detection rate of pulmonary nodules has markedly risen [1, 2], with approximately 40% exhibiting ground‐glass nodules (GGNs) on imaging [3]. The pathological spectrum of GGNs encompasses atypical adenomatous hyperplasia (AAH), adenocarcinoma in situ (AIS), minimally invasive adenocarcinoma (MIA), and invasive adenocarcinoma (IAC). In the 2021 fifth edition of the World Health Organization (WHO) classification of thoracic tumors, AAH and AIS were reclassified as adenomatous precursor lesions and excluded from the category of malignant pulmonary neoplasms [4]. MIA is defined as a solitary adenocarcinoma measuring ≤ 3 cm in greatest dimension, predominantly exhibiting a lepidic growth pattern and having an invasive component ≤ 5 mm [5, 6, 7]. MIA is associated with an excellent prognosis, with 5‐year postoperative survival rates approaching 100% [8]. For AAH/AIS and MIA, regular surveillance is generally recommended to determine optimal timing for surgical intervention. In contrast, IAC typically requires prompt surgical treatment due to its more aggressive behavior. Therefore, early identification of the invasiveness of GGNs is critical for guiding clinical decision‐making.

Conventional CT‐based diagnosis primarily relies on the radiologist's subjective assessment of morphological features of pulmonary nodules, such as lobulation, cavitation, and degree of enhancement [9, 10]. However, substantial overlap in CT characteristics exists among nodules with varying degrees of invasiveness, which makes it challenging to accurately differentiate their invasive potential based solely on conventional morphological criteria. Radiomics, an emerging field that converts medical images into high‐dimensional, mineable quantitative features, has facilitated the development of numerous predictive models based on imaging data [11, 12]. Previous studies have demonstrated that radiomics‐based models can enhance the accuracy of invasiveness prediction in pulmonary nodules [13, 14]. Nevertheless, the complexity of feature extraction and processing workflows hampers the widespread clinical implementation of radiomics technologies.

The integration of artificial intelligence (AI) with radiomics has transformed thoracic imaging by enabling high‐dimensional, quantitative analysis of pulmonary nodules [15]. Through automated extraction of CT‐derived features such as density, volume, entropy, and skewness, deep learning frameworks offer objective, reproducible insights into the biological behavior of nodules. This paradigm shift is redefining diagnostic workflows, establishing AI‐extracted CT radiomic features as a key element of precision imaging and clinical decision‐making. Compared with traditional radiomics, AI‐extracted CT radiomic features present distinct advantages: (i) they do not require complex post‐processing workflows and can be directly incorporated into routine CT reports; and (ii) their standardized definitions facilitate cross‐center comparisons, making them particularly suitable for use in primary care settings.

In recent years, machine learning (ML) techniques have demonstrated distinct advantages in the field of radiomics [16]. Algorithms such as random forest (RF) and support vector machine (SVM) have shown strong capabilities in efficiently handling high‐dimensional, small‐sample imaging data [17, 18], while also addressing issues of multicollinearity that commonly affect traditional logistic regression models.

In this study, we developed predictive models to assess the invasiveness of GGNs using AI‐extracted CT radiomic features in combination with multiple ML algorithms. Furthermore, we employed the Shapley Additive Explanations (SHAP) method to visualize and interpret key predictive factors, enabling accurate stratification of pulmonary nodule pathology.

## 2 Methods

### 2.1 Patient Cohort

Patients were retrospectively enrolled from two affiliated medical centers of the Third Affiliated Hospital of Sun Yat‐sen University, including the Lingnan Campus and the Tianhe Campus, between January 2024 and January 2025. Inclusion criteria were: (i) thin‐slice chest CT performed within 1 month before surgery with clearly identifiable GGNs on lung window settings; (ii) maximum lesion diameter < 3 cm; and (iii) postoperative histological diagnosis of AAH/AIS, MIA, or IAC. Exclusion criteria included: (i) incomplete clinical records; (ii) receipt of neoadjuvant chemotherapy, radiotherapy, immunotherapy, or targeted therapy prior to surgery; and (iii) poor CT image quality due to artifacts or insufficient lesion visibility. Patient selection flowchart was shown in Figure S1.

This study was approved by the Ethics Committee of the Third Affiliated Hospital of Sun Yat‐sen University (Approval Number: 2025‐059‐01) and conducted in accordance with the Declaration of Helsinki. The requirement for informed consent was waived due to the retrospective nature of the study.

### 2.2 Sample Size Calculation

In this study, we planned to include four predictive features in the final machine learning model. According to established empirical guidelines in machine learning, the recommended minimum sample size is approximately 100 times the number of predictive features to ensure adequate model stability and generalizability [19]. Based on this rule of thumb, at least 400 cases were required.

To satisfy this requirement, we retrospectively enrolled 285 patients from the Lingnan Campus of the Third Affiliated Hospital of Sun Yat‐sen University and supplemented the dataset with an additional 210 patients from the Tianhe Campus during the same study period. In total, 495 patients were included in the analysis, which provided sufficient statistical power for robust model development and validation.

### 2.3 Data Acquisition

DICOM‐format chest CT images were imported into the InferRead CT Lung AI platform (Infervision), which automatically segmented the target nodule region. All segmentations were reviewed and confirmed by experienced radiologists. After confirmation, quantitative radiomic features of the pulmonary nodules were extracted. The parameters included: nodule volume, surface area, 3D maximum cross‐sectional area, weight, distance to pleura, 3D long axis diameter, transverse diameter, maximum, minimum, mean, median, and standard deviation of CT values, solid component proportion, compactness, sphericity, kurtosis, skewness, energy, and entropy. In addition, peripheral blood biomarkers relevant to lung cancer were measured, including carcinoembryonic antigen (CEA), squamous cell carcinoma antigen (SCC), neuron‐specific enolase (NSE), and cytokeratin 19 fragment (CYFRA 21‐1).

### 2.4 ML Models Construction

A two‐stage feature selection strategy was employed to enhance model robustness. First, the Boruta algorithm—a wrapper‐based method leveraging shadow features generated via permutation of the original feature matrix—was used to identify statistically relevant predictors. A random forest classifier (n_estimators = 500) was trained across 100 iterations, with a significance threshold set at *p* < 0.01 (two‐sided permutation test). Only features with importance scores consistently exceeding those of their permuted counterparts were retained. In the second stage, the feature space was further refined using least absolute shrinkage and selection operator (LASSO) regression with 10‐fold cross‐validation. The optimal regularization parameter (λ) was determined by minimizing the binomial deviance via coordinate descent. Features with non‐zero coefficients were retained for final model construction, ensuring both sparsity and biological interpretability. The selected features were subsequently applied to seven distinct ML algorithms—logistic regression (LR), random forest (RF), extreme gradient boosting (XGBoost), neural network (NN), naive Bayes (NB), gradient boosting machine (GBM), and support vector machine (SVM), to evaluate predictive performance and generalizability across modeling frameworks. Hyperparameters for all models were optimized using Bayesian optimization with Gaussian process priors (GPyOpt library), implemented via five‐fold cross‐validation within the training cohort.

### 2.5 Statistical Analysis

Categorical variables were summarized as counts and percentages. Continuous variables were expressed as mean ± standard deviation (SD) if normally distributed; otherwise, they were reported as median and interquartile range (IQR; 25th–75th percentile). Comparisons of categorical variables between groups were performed using the Pearson *χ* ^2^ test or Fisher's exact test. For continuous variables, independent‐sample Student's *t*‐tests were used for normally distributed data, while non‐normally distributed data were compared using the Mann–Whitney U test.

Model performance was systematically assessed using a predefined composite metric framework, including the area under the receiver operating characteristic curve (AUC‐ROC), accuracy, positive and negative predictive values (PPV/NPV), sensitivity, specificity, and F1 score. Clinical utility was quantified via decision curve analysis (DCA) by calculating net benefit across a range of threshold probabilities. Bootstrapping with 1000 iterations was used to estimate 95% confidence intervals (95% CI) for all metrics. The model yielding the highest AUC‐ROC was selected as the optimal classifier. To interpret feature contributions, a SHAP analysis was implemented using the KernelExplainer algorithm, enabling global ranking of feature importance.

All statistical analyses were performed using R software (version 4.3.0; R Foundation for Statistical Computing). A two‐sided *p*‐value < 0.05 was considered statistically significant.

## 3 Results

### 3.1 Baseline Analysis

A total of 495 patients were included, with 266 in the AAH/AIS/MIA group and 229 in the IAC group. Compared to the AAH/AIS/MIA group, patients in the IAC group were significantly older (median 60 vs. 51 years) and more likely to be male (51.1% vs. 36.1%) and have a history of smoking (25.6% vs. 13.9%). Serum biomarker levels, including CEA (*p* < 0.001), SCC (*p* = 0.038), and CYFRA 21‐1 (*p* < 0.001), were significantly elevated in the IAC group, whereas NSE levels did not differ significantly between groups (*p* = 0.351). Radiologically, IAC exhibited markedly greater volume (1396 vs. 248 mm^3^, *p* < 0.001), surface area (987 vs. 257 mm^2^, *p* < 0.001), 3D maximum area (181 vs. 56 mm^2^, *p* < 0.001), along with shorter distances to the pleura (3 vs. 9 mm, *p* < 0.001), suggesting more aggressive and invasive growth. CT values were significantly higher in the IAC group, with increased maximum (*p* < 0.001), minimum (*p* < 0.001), mean (*p* < 0.001), and median (*p* < 0.001) hounsfield units, as well as a substantially larger solid component proportion (64% vs. 8%, *p* < 0.001). Textural and morphologic differences were also apparent: IAC had lower compactness and sphericity (*p* < 0.001), along with reduced kurtosis and skewness (*p* < 0.001), and elevated energy and entropy (*p* < 0.001), indicating greater internal heterogeneity and architectural complexity. Baseline clinical, serological, and radiological characteristics of patients with AAH/AIS/MIA and IAC were shown in Table 1.

**TABLE 1:** Patient demographics and baseline characteristics.

Characteristic	AAH/AIS/MIA group *N* = 266	IAC group *N* = 229	*p*
Sex			
Female	170 (63.9%)	112 (48.9%)	0.001^***^
Male	96 (36.1%)	117 (51.1%)	
Age	51 (18, 81)	60 (24, 84)	< 0.001^***^
Primary site			
Upper lobe	156 (58.6%)	125 (54.6%)	0.511
Middle lobe	21 (7.9%)	24 (10.5%)	
Lower lobe	89 (33.5%)	80 (34.9%)	
Smoking history			
No	229 (86.1%)	198 (74.4%)	0.001^***^
Yes	37 (13.9%)	68 (25.6%)	
Serum biomarkers			
CEA	1.53 (1.04, 2.46)	2.32 (1.53, 4.89)	< 0.001^***^
SCC	0.81 (0.41, 0.98)	0.82 (0.60, 1.13)	0.038^*^
NSE	10.43 (9.25, 15.68)	11.71 (10.34, 14.38)	0.351
CYFRA	2.32 (1.41, 3.62)	3.12 (2.53, 4.17)	< 0.001^***^
Radiologic features			
Volume	248 (134, 441)	1396 (521, 3291)	< 0.001^***^
Surface area	257 (183, 464)	987 (416, 1712)	< 0.001^***^
3D maximum area	56 (38, 97)	181 (84, 328)	< 0.001^***^
Weight	127 (81, 226)	1314 (3663202)	< 0.001^***^
Distance from nodule to pleura	9 (2, 16)	3 (0, 11)	< 0.001^***^
Transverse diameter	11 (7, 19)	17 (12, 21)	< 0.001^***^
3D long diameter	14 (10, 21)	22 (14, 32)	< 0.001^***^
Maximum CT value	291 (80, 506)	549 (317, 726)	< 0.001^***^
Minimum CT value	−741 (−818, −692)	−520 (−726, −271)	< 0.001^***^
Mean CT value	−513 (−618, −429)	−77 (−381, 29)	< 0.001^***^
Median of CT value	−558 (−657, −462)	−59 (−381, 29)	< 0.001^***^
Standard deviation of CT value	181 (144, 236)	173 (148, 232)	0.639
Proportion of solid components	8 (1, 16)	64 (20, 92)	< 0.001^***^
Compactness	0.041 (0.033, 0.059)	0.039 (0.025, 0.041)	< 0.001^***^
Sphericity	0.79 (0.72, 0.88)	0.71 (0.61, 0.78)	< 0.001^***^
Kurtosis	0.48 (−0.48, 0.99)	−0.83 (−1.00, −0.68)	< 0.001^***^
Skewness	0.72 (0.49, 0.99)	0.15 (0.11, 0.34)	< 0.001^***^
Energy	2.49 (1.48, 4.86)	4.48 (2.54, 8.23)	< 0.001^***^
Entropy	5.17 (4.63, 5.42)	5.49 (5.10, 5.67)	< 0.001^***^

*p < 0.05, ***p < 0.001.

### 3.2 Model Building and Validation

The populations from the Lingnan Campus of the Third Affiliated Hospital of Sun Yat‐sen University were randomly divided into a training cohort and an internal validation cohort at an 8:2 ratio. The patients from the Tianhe Campus of the Third Affiliated Hospital of Sun Yat‐sen University were included in the external validation cohort. The characteristic baseline of patients in the three cohorts was summarized in Table 2. Clinical characteristics, including age (*p* = 0.601), sex (*p* = 0.190), laterality (*p* = 0.428), primary site (*p* = 0.230), smoking history (*p* = 0.992), and pathology (*p* = 0.324) were well balanced between the three groups, ensuring comparability for subsequent model development and evaluation.

**TABLE 2:** Characteristic baseline of patients in train, internal validation and external validation cohort.

Characteristic	Training cohort *N* = 229	Internal validation cohort *N* = 56	External validation cohort *N* = 210	*p*
Age	57 (46, 65)	54 (44, 65)	57 (25, 81)	0.601
Sex				
Female	127 (55.5%)	27 (48.2%)	128 (60.9%)	0.190
Male	102 (44.5%)	29 (51.8%)	82 (39.1%)	
Laterality				
Left	108 (47.2%)	21 (37.5%)	95 (45.2%)	0.428
Right	121 (52.8%)	35 (62.5%)	115 (84.8%)	
Primary site				
Upper lobe	125 (54.6%)	32 (57.1%)	124 (59.0%)	0.230
Middle lobe	25 (10.9%)	1 (1.8%)	19 (9.0%)	
Lower lobe	79 (34.5%)	23 (41.1%)	67 (32.0%)	
Smoking history				
No	181 (79.0%)	44 (78.6%)	165 (78.6%)	0.992
Yes	48 (21.0%)	12 (21.4%)	45 (21.4%)	
Pathology				
AAH/AIS/MIA	115 (50.2%)	33 (58.9%)	118 (56.2%)	0.324
IAC	114 (49.8%)	23 (41.1%)	92 (43.8%)	

**FIGURE 1:**
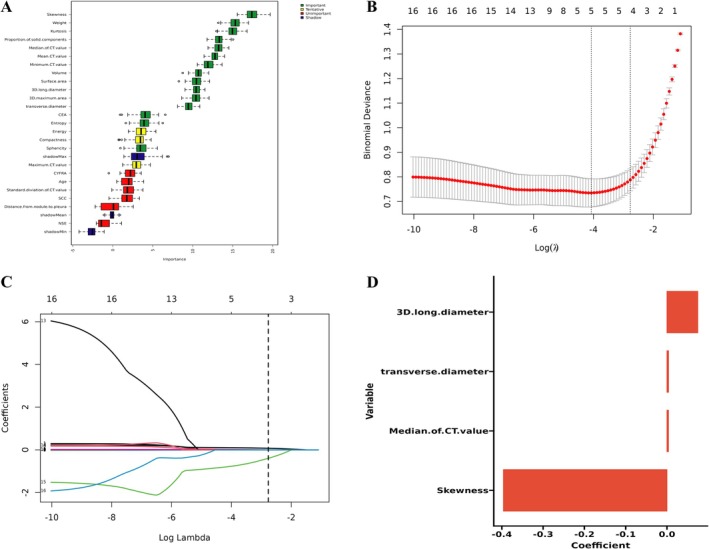
The feature selection process. (A) Ranking of features for predicting invasiveness of pulmonary nodule by Boruta algorithm. The plot demonstrates boxplot of important attributes in color green, tentative attributes in yellow, non‐important attributes in red, and shadow attributes in blue box, respectively. The vertical axis lists the name of each variable, and the horizontal axis is the *Z*‐value. (B) Features determined by LASSO analysis (*n* = 4). (C) LASSO Coefficient distribution map‐LASSO coefficient distribution of all features. (D) Coefficients for the four key features in Lasso model.

A total of 19 radiological features and five clinical variables were initially extracted. Feature selection was performed using the Boruta algorithm, which identified 15 predictors with high relevance: skewness, weight, kurtosis, proportion of solid components, median CT value, mean CT value, minimum CT value, volume, surface area, 3D long diameter, 3D maximum area, transverse diameter, CEA, entropy, and sphericity. These 15 features were subsequently entered into a LASSO regression model for dimensionality reduction. Four variables with non‐zero coefficients were retained in the final model: 3D long diameter (coefficient = 0.075), transverse diameter (coefficient = 0.004), median CT value (coefficient = 0.004), and skewness (coefficient = −0.396). The feature selection process is illustrated in Figure 1.

**FIGURE 2:**
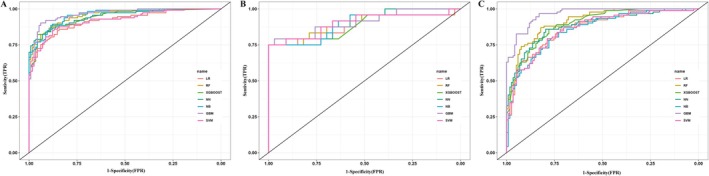
ROC curves for predicting pathological invasiveness of pulmonary ground‐glass nodules using different machine learning algorithms. (A) ROC curves for training cohort. (B) ROC curves for internal validation cohort. (C) ROC curves for external validation cohort.

Using the four selected features, we developed seven ML models to predict the pathological invasiveness of GGNs. As shown in Figure 2, receiver operating characteristic (ROC) curves were generated to evaluate model discrimination. In the training cohort, the AUC‐ROC for each model was as follows: LR (0.919, 95% CI: 0.885–0.995), RF (0.955, 95% CI: 0.932–0.978), XGBoost (0.942, 95% CI: 0.914–0.971), GBM (0.965, 95% CI: 0.944–0.985), SVM (0.921, 95% CI: 0.886–0.956), NB (0.957, 95% CI: 0.934–0.979), and NN (0.947, 95% CI: 0.920–0.974). In the internal validation cohort, the AUC‐ROC was as follows: LR (0.880, 95% CI: 0.776–0.984), RF (0.904, 95% CI: 0.821–0.987), XGBoost (0.887, 95% CI: 0.796–0.978), GBM (0.908, 95% CI: 0.824–0.992), SVM (0.902, 95% CI: 0.809–0.994), NB (0.890, 95% CI: 0.794–0.987), and NN (0.894, 95% CI: 0.807–0.981). Similarly, in the external validation cohort, all models maintained robust predictive performance: LR (0.865, 95% CI: 0.816–0.914), RF (0.915, 95% CI: 0.878–0.951), XGBoost (0.897, 95% CI: 0.856–0.937), GBM (0.965, 95% CI: 0.945–0.984), SVM (0.863, 95% CI: 0.813–0.912), NB (0.858, 95% CI: 0.808–0.907), and NN (0.884, 95% CI: 0.838–0.931).

**FIGURE 3:**
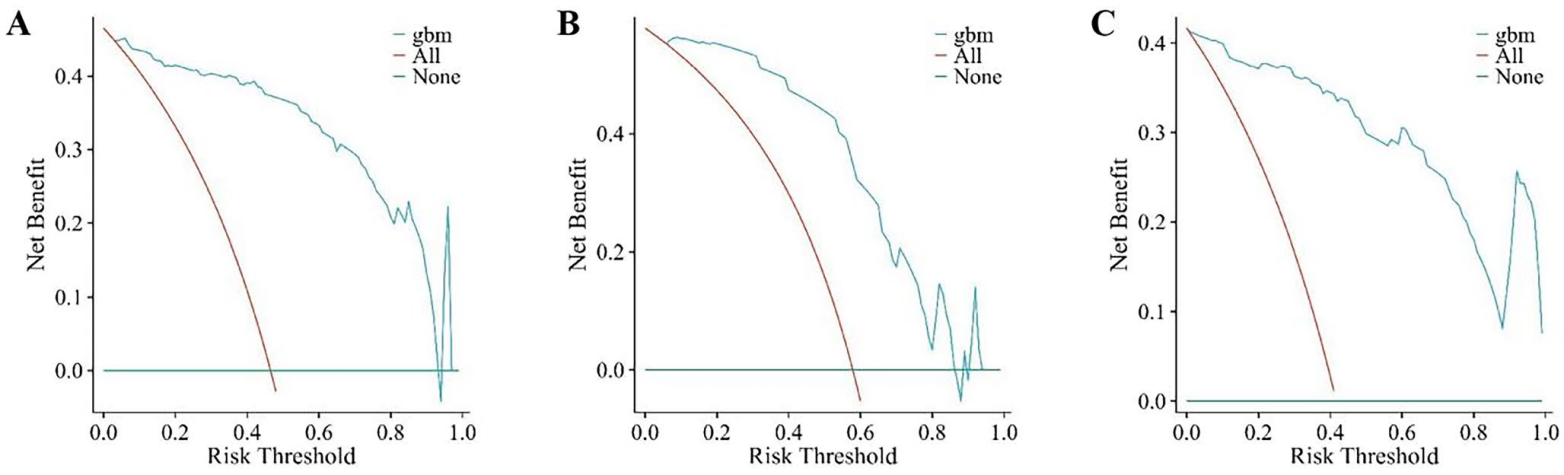
Decision curve analysis of the GBM model of the training cohort (A), internal validation cohort (B), and external validation cohort (C). The vertical axis is the net benefit after intervention; the horizontal axis is the threshold.

The clinical applicability of the GBM model was assessed using DCA. As shown in Figure 3, the GBM model demonstrated favorable net clinical benefit across the training cohort, internal validation cohort, and external validation cohort. Comprehensive performance metrics, including accuracy, PPV, NPV, recall, specificity, and F1 score, were summarized in Tables 3, 4, 5. In the training cohort, the GBM model achieved an accuracy of 0.9123, PPV of 0.9266, NPV of 0.8992, recall of 0.8938, specificity of 0.9304, and F1 score of 0.9099. In the internal validation cohort, performance remained robust with an accuracy of 0.8772, PPV of 0.9474, NPV of 0.8421, recall of 0.7500, specificity of 0.9697, and F1 score of 0.8372. In the external validation cohort, the GBM model still achieved high predictive performance, with an accuracy of 0.8810, a positive predictive value (PPV) of 0.8073, a negative predictive value (NPV) of 0.9604, a recall (sensitivity) of 0.9565, a specificity of 0.8220, and an F1 score of 0.8756.

**TABLE 3:** Evaluation of the prediction performance of seven predictive models in the training cohort.

	Accuracy	PPV	NPV	Recall	Specific	F‐measure
LR	0.8421	0.8348	0.8496	0.8496	0.8348	0.8421
RF	0.8728	0.8750	0.8707	0.8673	0.8783	0.8711
XGboost	0.8816	0.9388	0.8385	0.8142	0.9478	0.8720
NN	0.8684	0.8952	0.8455	0.8319	0.9043	0.8624
NB	0.8772	0.8696	0.8850	0.8850	0.8696	0.8772
GBM	0.9123	0.9266	0.8992	0.8938	0.9304	0.9099
SVM	0.8553	0.8571	0.8534	0.8496	0.8609	0.8533

**TABLE 4:** Evaluation of the prediction performance of seven predictive models in the internal validation cohort.

	Accuracy	PPV	NPV	Recall	Specific	F‐measure
LR	0.8070	0.7826	0.8235	0.7500	0.8485	0.7660
RF	0.8421	0.8571	0.8333	0.7500	0.9091	0.8000
XGboost	0.8596	0.9000	0.8378	0.7500	0.9394	0.8182
NN	0.8246	0.8182	0.8286	0.7500	0.8788	0.7826
NB	0.7719	1.0000	0.7174	0.4583	1.0000	0.6286
GBM	0.8772	0.9474	0.8421	0.7500	0.9697	0.8372
SVM	0.8772	0.9474	0.8421	0.7500	0.9697	0.8372

**TABLE 5:** Evaluation of the prediction performance of seven predictive models in the external validation cohort.

	Accuracy	PPV	NPV	Recall	Specific	F‐measure
LR	0.7762	0.7027	0.8586	0.8478	0.7203	0.7685
RF	0.8333	0.7822	0.8807	0.8587	0.8136	0.8187
XGboost	0.8095	0.7500	0.8679	0.8478	0.7797	0.7959
NN	0.8143	0.7573	0.9692	0.8478	0.7881	0.8000
NB	0.7714	0.7157	0.8241	0.7935	0.7542	0.7526
GBM	0.8810	0.8073	0.9604	0.9565	0.8220	0.8756
SVM	0.7810	0.7347	0.8214	0.7826	0.7797	0.7579

### 3.3 Feature Importance

**FIGURE 4:**
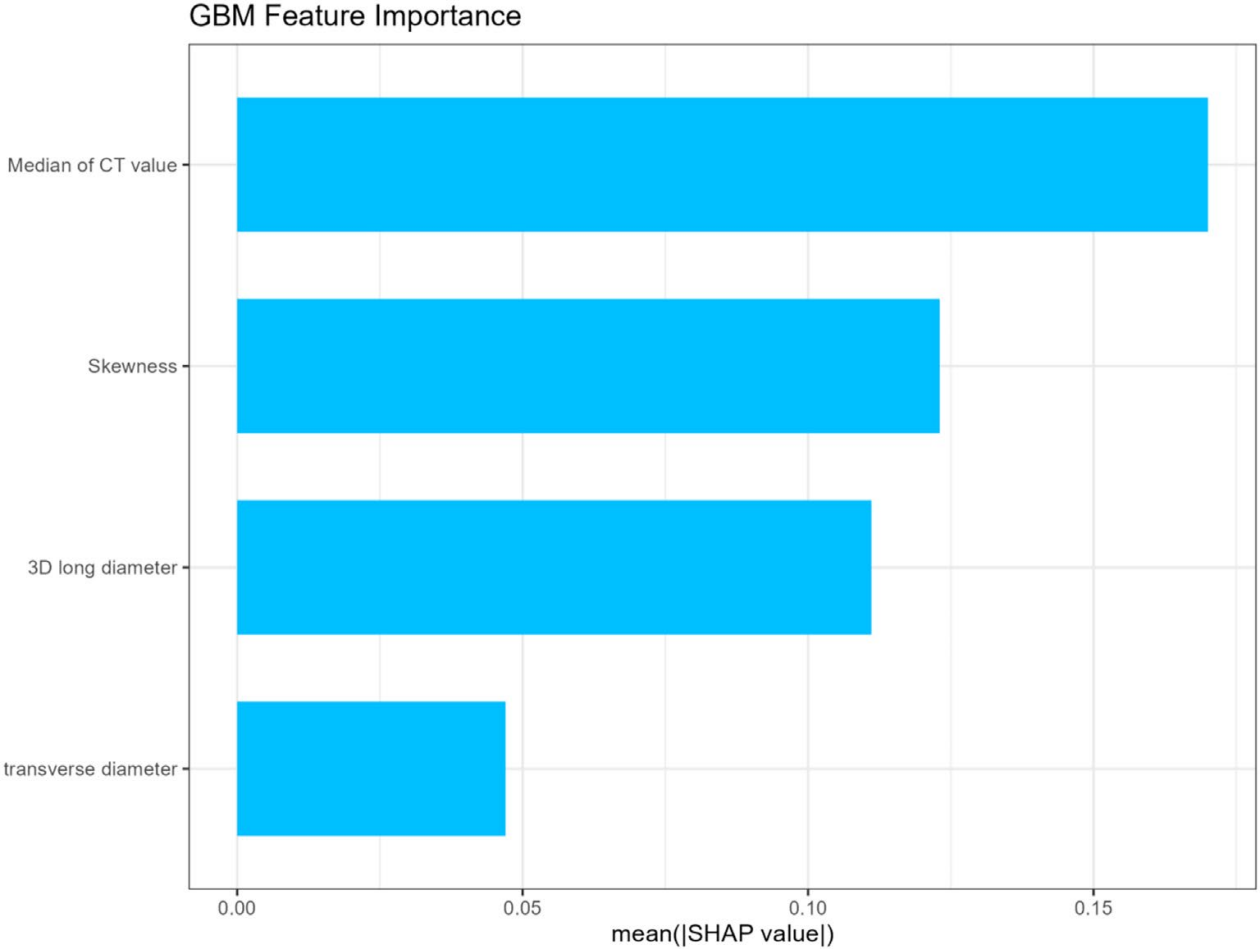
The mean SHAP values of features for the GBM model. The horizontal axis represents the average SHAP value, and the vertical axis represents the predictor in the GBM model.

**FIGURE 5:**
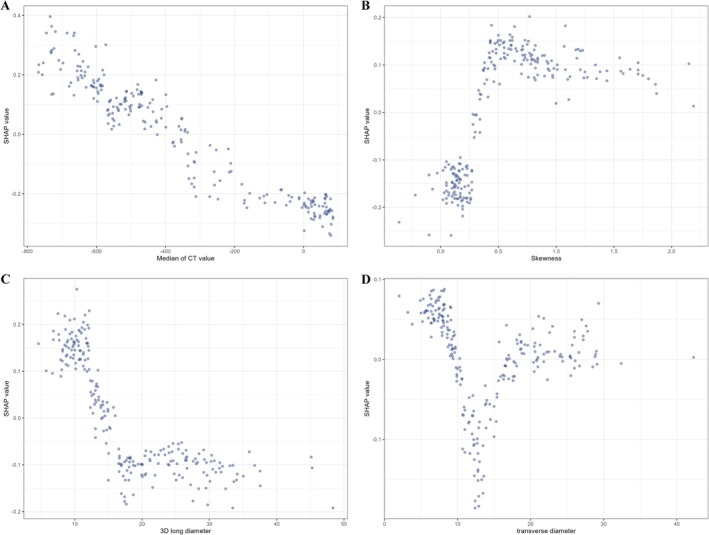
Single feature SHAP dependency graph, the horizontal axis represents the value range of a single feature, the vertical axis represents the SHAP value of the feature, and the scattered points represent each sample.

To elucidate the key drivers underlying the predictive performance of the GBM model, SHAP analysis was performed to quantify the contribution of each input variable to model output. As shown in Figures 4 and 5, the mean SHAP values indicated that the median CT value exerted the strongest influence on model predictions, followed by skewness, then the 3D long diameter, and transverse diameter.

## 4 Discussion

This study constructed a powerful ML model based on the AI‐extracted CT radiomic features to predict the invasiveness of pulmonary nodules and provide guidance for surgical decisions. Among the evaluated models, the GBM achieved the highest discriminative performance (AUC: 0.965 in training cohort, 0.908 in internal validation cohort, 0.965 in external cohort). Our findings offer two key contributions to pulmonary nodule risk stratification: (i) development of a streamlined, four‐feature model that balances predictive accuracy with interpretability. (ii) identification of median CT value and skewness as dominant imaging predictors of invasiveness.

The deep integration of AI with high‐resolution computed tomography (HRCT) has enabled clinicians to extract micro‐level imaging features beyond the limits of conventional visual interpretation, markedly improving the predictive accuracy for malignancy in pulmonary nodules [20, 21, 22]. However, therapeutic strategies diverge markedly based on the degree of tumor invasiveness. Preinvasive lesions, such as AAH/AIS or MIA, typically require limited wedge resection. In contrast, IAC often requires segmentectomy or lobectomy, depending on factors such as nodule size (> 2 cm) and proportion of solid components (> 50%) [23]. In current clinical practice, intraoperative frozen section analysis is frequently used to guide the extent of resection. Yet its diagnostic concordance with final paraffin‐embedded histopathology remains suboptimal (*κ* = 0.45–0.65) [24, 25], contributing to both overtreatment and undertreatment. These limitations underscore the critical need for accurate, noninvasive preoperative assessment of nodule invasiveness to guide surgical decision‐making.

This study identified a strong positive correlation between the median CT value of pulmonary nodules and their pathological invasiveness, with this feature contributing most significantly to model performance. CT value is closely associated with the internal composition of tumors, including cellular density, fibrosis, and necrosis. Preinvasive lesions such as AAH/AIS and MIA are characterized histologically by lepidic growth along intact alveolar walls, preserving aerated spaces and resulting in lower CT value [26]. In contrast, IAC typically exhibits solid components with densely packed tumor cells, fibrosis, or necrotic foci [26], contributing to markedly higher CT value. Thus, CT value may serve as a surrogate marker for histologic aggressiveness. These findings are consistent with prior studies by Kitazawa et al. [27] and Yang et al. [28]. Importantly, the distribution of solid components within nodules is often heterogeneous. Compared to maximum or mean CT value, the median CT value demonstrates greater robustness to outliers and more reliably reflects the representative tissue density of the lesion's core architecture.

In radiomics, skewness quantifies the asymmetry of CT values distribution and serves as a critical metric for capturing intratumoral heterogeneity. Its diagnostic relevance in pulmonary nodule characterization has gained increasing attention [29, 30]. A positive skewness indicates a predominance of low‐density voxels (e.g., ground‐glass components), whereas negative skewness reflects a distribution tailing toward higher‐density regions, such as solid components or calcifications. Yoshiyasu et al. [31] previously observed that declining skewness over time correlates with increased nodule invasiveness. Although prior investigations have suggested an association between skewness and tumor invasiveness, their findings were constrained by limited sample sizes. In our study, this relationship was validated in a larger patient cohort, demonstrating that IAC exhibit significantly lower skewness values than AAH/AIS/MIA (median: 0.15 vs. 0.72; *p* < 0.001), reflecting a shift in CT values toward higher‐density regions. This pattern is closely associated with aggressive histopathologic features such as increased tumor cellularity and stromal fibrosis.

Maximum nodule diameter is a well‐established indicator of tumor invasiveness. Larger diameters are associated with greater tumor burden and a higher likelihood of stromal invasion, vascular encroachment, or lymphatic spread. In a study by Chenggong et al. [32] involving 74 pulmonary nodules, lesion diameter was identified as a significant predictor of invasiveness. Similarly, findings by Peng Zhou et al. [33] further supported this result. In our study, we evaluated nodule size from both two‐dimensional (2D) and three‐dimensional (3D) perspectives. Both 2D and 3D long‐axis diameters emerged as independent predictors of invasiveness. Notably, the 3D long diameter contributed more substantially to model performance than its 2D transverse diameter. These findings suggest that volumetric measurement of nodule size in 3D space offers superior discriminatory power and may be preferable in assessing malignant potential. This may be because 3D diameter reflects tumor geometry more accurately than traditional 2D measurements and can capture subtle extensions along the oblique surface, thus more accurately predicting aggressive growth patterns.

Collectively, these radiomic features reflect key biological hallmarks of malignancy, including increased cellularity, architectural disruption, stromal remodeling, and heterogeneity in the tumor microenvironment. Their ability to noninvasively infer such characteristics underscores the biological interpretability of AI‐based models and supports their utility in guiding individualized management decisions.

Finally, we implemented multiple ML algorithms using the selected features, achieving strong predictive performance across all models, with AUCs ranging from 0.858 to 0.965 in both the training and validation cohorts. While previous studies have developed CT‐based predictive models for tumor invasiveness, most employed conventional logistic regression. In contrast, our study utilized a two‐stage feature selection pipeline—Boruta followed by LASSO regression—which effectively reduced overfitting risks inherent in high‐dimensional radiomics. This approach compressed the initial 24 features to 4 while preserving 92% of the predictive variance. Compared to traditional univariate filtering or stepwise regression [34, 35, 36, 37], which often overlook interaction effects critical to invasiveness, this strategy better captured multivariate dependencies. Among seven ML algorithms evaluated, the GBM model demonstrated the highest predictive performance. Moreover, the GBM model showed superior net clinical benefit in DCA analysis across a wide range of threshold probabilities, indicating its utility in surgical decision‐making.

Importantly, our model relies on four interpretable features—median CT attenuation, skewness3D long diameter, and transverse diameter—addressing the “black‐box” criticism of AI models [38, 39]. This interpretability allows clinicians to audit model behavior through radiologic reasoning, thus enhancing trust and clinical applicability.

Despite rigorous internal validation, our study has limitations. First, the retrospective single‐center design introduces selection bias. Prospective multicenter trials are needed to validate feature generalizability across diverse CT scanners and populations. Second, while SHAP values elucidated model behavior, deeper biological validation is warranted. Third, this study developed a binary classification model to distinguish IAC from preinvasive or minimally invasive lesions. However, it did not further differentiate between AAH/AIS and MIA. Future work should focus on developing a multiclass classification model capable of discriminating among AAH/AIS, MIA, and IAC, which would offer more granular guidance for surgical planning and risk stratification.

## 5 Conclusion

This study demonstrates the feasibility of integrating AI‐extracted CT radiomic features with ML to predict the pathological invasiveness of pulmonary nodules. The resulting model provides accurate, noninvasive risk stratification and may serve as a valuable tool to inform personalized clinical management strategies for patients with indeterminate lung nodules.

## Author Contributions

All authors contributed to the study conception and design. The first draft of the manuscript was written by Guozhen Yang. The material preparation was performed by Yuanheng Huang. The data collection was performed by Huiguo Chen. The analysis was performed by Weibin Wu. The interpretation was performed by Yonghui Wu and Jiannan Xu. The article revision was done by Kai Zhang and Xiaojun LI. The study conception and design was performed by Jian Zhang. All authors read and approved the final manuscript.

## Ethics Statement

This study was conducted in accordance with the ethical principles of the Declaration of Helsinki (for research on human subjects) and was approved by the Ethics Committee of the Third Affiliated Hospital of Sun Yat‐sen University (Approval Number: 2025–059‐01). Informed consent was waived due to the retrospective nature of the analysis.

## Consent

All authors agreed to publish this article.

## Conflicts of Interest

The authors declare no conflicts of interest.

## Supporting information


**Figure S1.** Study flowchart of the enrolled patients. AAH, atypical adenomatous hyperplasia; AIS, adenocarcinoma in situ; GGNs, pure ground glass nodules; MIA, minimally invasive adenocarcinoma.

## Data Availability

Research data are not shared.
