# Supplementary material for: Machine Learning Model for Predicting Pathological Invasiveness of Pulmonary Ground‐Glass Nodules Based on AI‐Extracted Radiomic Features
Source: Thorac Cancer. 2025 Jul 31;16(15):e70128. doi: 10.1111/1759-7714.70128 (PMC12313823; doi:10.1111/1759-7714.70128)
Supplement: Supplementary file 1 — Figure S1. Study flowchart of the enrolled patients. AAH, atypical adenomatous hyperplasia; AIS, adenocarcinoma in situ; GGNs, pure ground glass nodules; MIA, minimally invasive adenocarcinoma. [file TCA-16-e70128-s001.docx]

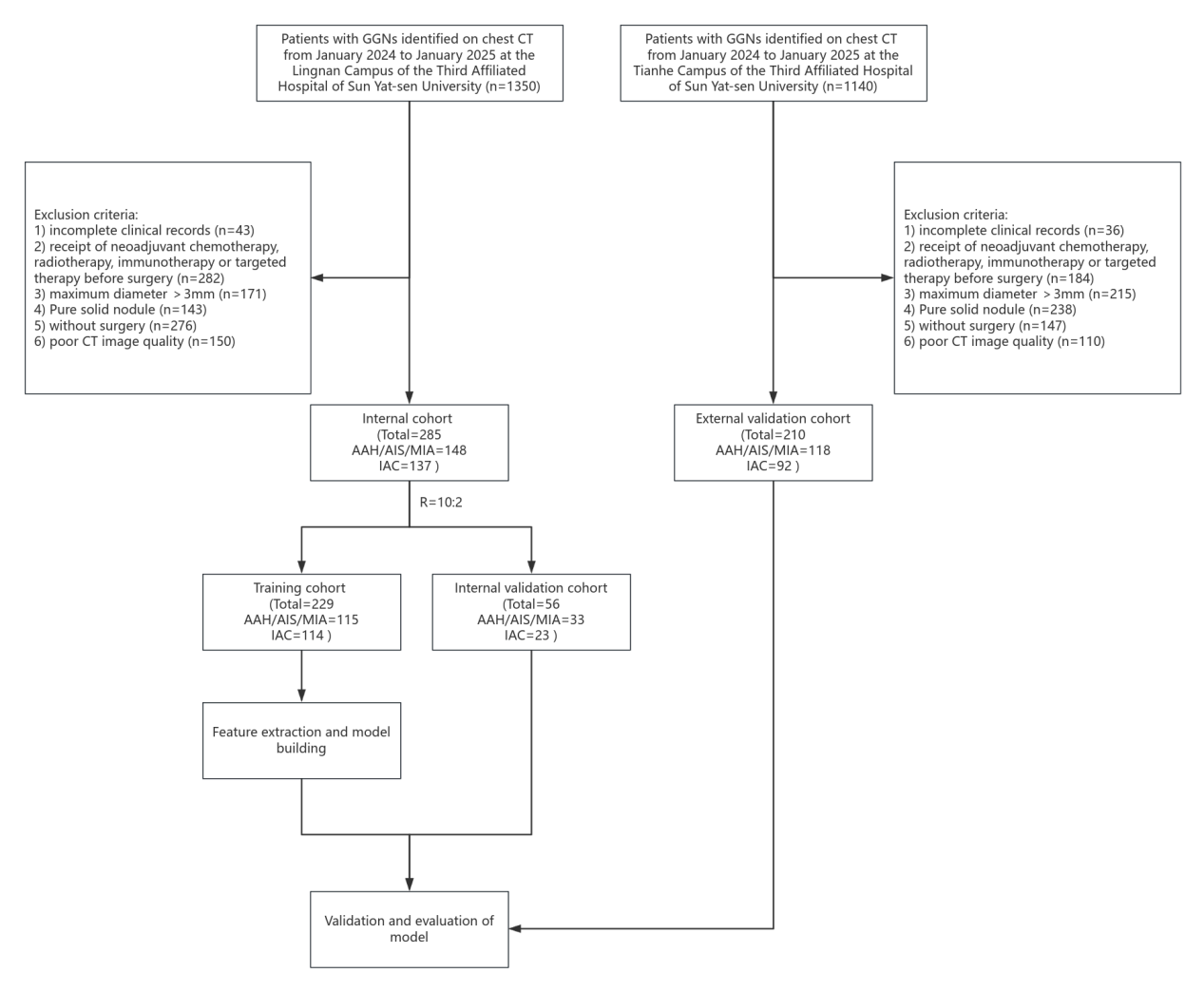


**Supplementary figure 1.** Study flowchart of the enrolled patients. AAH, atypical adenomatous hyperplasia; AIS, adenocarcinoma in situ; MIA, minimally; invasive adenocarcinoma; GGNs, pure ground glass nodules.
